# New Anæsthetics

**Published:** 1870-09

**Authors:** 


### ARTICLE VI.

### *New Anæsthetics.*

BROMOFORM, BROMAL AND IODAL.

*Bromoform* possesses analogous physiological properties to *Chloroform*, and *Bromal* and *Iodal* produce in the animal organism similar effects to those caused by *Chloral.*

To prepare *Bromoform*, we make at first the *Bromal*, and combine it with the base. The formula is:

$$\underbrace{C^2HBr^3O}_{\text{Bromal}} + \underbrace{KHO}_{\text{Potassa}} = \underbrace{CHBr^3}_{\text{Bromoform}} + \underbrace{CHKO^2}_{\text{Formate of Potash}}$$

The liquid obtained by distillation between 50 and 65°, presents chemical and organoleptical properties perfectly analogous to *Chloroform*, so that it could be mistaken for it with the difference that *Chloroform*, mixed with *Iodine*, gives an azure light violet color, whereas *Bromoform*, mixed with the same metal, gives a most beautiful red carmine. A dog was allowed to inhale a small quantity of *Bromoform*, which produced complete anæsthesia. He could be pinched and pricked without feeling it The pupils were enormously distended. *Bromoform* produces anæsthesia when given in very small quantities, without producing a profound or a dangerous sleep.

*Bromal* differs from *Chloroform* only in that, that the *Chlor.* is replaced by the *Brom.* Its formula is $C^2HBrO$, and to chrystallize it we must make the hydrate: $C^2HBrO + H^2O$. During all the time Dr. R. experimented with it, there was a great discharge from the eyes and nose.

*Iodal* is prepared by mixing the *Iodine* with a quantity of alcohol and azotic acid, producing thus, also, *Iodoform* and a formiate. Like *Bromal*, it effects lachrymation. 5–6 grains injected into the rectum, produced anæsthesia, followed by convulsions and death. The blood was black, the muscles more red than usual; the mesenterium, the brain, and the medulla spinalis congested. The breath of the dog smelled strongly of *Iodal*, proving that it became only partially decomposed in the body.—*Dr. Rabuteau in L'ArtDentaire.*

